# Comparative analysis of adenosine 1 receptor expression and function in hippocampal and hypothalamic neurons

**DOI:** 10.1007/s00011-024-01980-8

**Published:** 2025-01-08

**Authors:** Lea Wegmann, Helmut L. Haas, Olga A. Sergeeva

**Affiliations:** 1 Medical Faculty and University Hospital, Institute of Neural and Sensory Physiology, Heinrich Heine University Düsseldorf, 40225 Düsseldorf, Germany; 2 Medical Faculty and University Hospital, Institute of Clinical Neurosciences and Medical Psychology, Heinrich Heine University Düsseldorf, 40225 Düsseldorf, Germany

**Keywords:** Tuberomamillary nucleus, Patch-clamp, Adenosine, Single-cell RT-PCR, Adenosine receptor 1, Histaminergic neurons

## Abstract

**Background:**

Adenosine, an ATP degradation product, is a sleep pressure factor. The adenosine 1 receptor (A1R) reports sleep need. Histaminergic neurons (HN) of the tuberomamillary nucleus (TMN) fire exclusively during wakefulness and promote arousal. All of them express GABA_A_ receptors and are inhibited by GABA. Does adenosine contribute to their silencing?

**Subjects and treatment:**

Responses to adenosine were studied in mouse brain slices and primary dissociated cultures. For HN identification single-cell (sc)RT-PCR, reporter protein and pharmacology were used. Hippocampal Dentate Gyrus granular layer cells (DGgc) were studied in parallel.

**Methods:**

Firing frequency was recorded in patch-clamp configuration or by microelectrode arrays. A1R-expression was studied by scRT-PCR and semiquantitative PCR.

**Results:**

Most DGgc were inhibited through A1R, detected with scRT-PCR in 7 out of 10 PDZd2-positive DGgc; all HN were A1R negative. One HN out of 25 was inhibited by adenosine. The A1R mRNA level in the hippocampus was 6 times higher than in the caudal (posterior) hypothalamus. Response to adenosine was weaker in hypothalamic compared to hippocampal cultures.

**Conclusions:**

Most HN are not inhibited by adenosine.

## Introduction

Adenosine, a degradation product of ATP, is a sleep pressure factor that plays an important role in homeostatic behavioural state control [1]. The adenosine 1 receptor (A1R) is most sensitive to adenosine and reports sleep need to the brain. Controversial data have been published during the last 18 years regarding hypothalamic histaminergic neurons (HN) [2–5], which control wakefulness [6]. Recent reviews describe a particular role of A1R on HN in the action of adenosine for sleep gating in rodents [7, 8], whereas we have neither seen inhibition of neuronal firing nor modulation of spontaneous inhibitory postsynaptic potentials (sIPSP) by adenosine in rat HN recorded in brain slices [2]. We analysed single-cell whole-transciptome data published recently [9, 10] and did not find A1R in any HN database. The “Hipposeq” study by Cembrowski et al. [11] reported that among adenosine receptors only the A1R is expressed by hippocampal dorsal Dentate Gyrus granular layer cells (DGgc), which can be identified by the expression of the cellular type marker PDZ domain containing 2 (PDZd2). We selected these cells as positive control. Moreover, a physiological role of postsynaptic A1R in hippocampal neurons is well characterized: it hyperpolarizes cells and suppresses epileptic seizures [12, 13]. To the best of our knowledge, no data exist showing inhibition of firing in response to adenosine in identified HN. In contrast, all HN express GABA_A_ receptors and are inhibited by GABA, which promotes slow-wave sleep and habituation to a novel environment [14–18]. We performed now electrophysiology and PCR to identify HN and DGgc, to study responses to adenosine and expression of A1R in mouse brain slices. Furthermore, we quantified A1R expression in hippocampal and posterior hypothalamic tissues and studied responses to adenosine in hippocampal (HPC) and caudal (posterior) hypothalamic (cHPT) primary dissociated cultures to determine the role of A1R for neuronal network activity of cultured HPC and cHPT neurons.

## Materials and methods

### Animals and slice electrophysiology

Juvenile and adult mice of both sexes expressing the fluorescent Tomato protein under control of the histidine decarboxylase promoter Tmt-HDC [19] as well as their parent lines (HDC-Cre and Rosa26-lox-STOP-lox-Tomato (Tmt)) and other available Cre-lines (further called “wild type”) were used to study responsiveness of histaminergic neurons (HN) and hippocampal Dentate Gyrus granular layer cells (DGgc) to adenosine and the adenosine 1 receptor antagonist DPCPX. All mice had a C57Bl6 genetic background; they were held at 12/12 h dark/light cycle, temperature 18–22 °C, 55% humidity and free access to water and food under specific pathogen-free conditions. All procedures were in compliance with the guidelines for the use of experimental animals, as given by the Directive 2010/63/EU of the European Parliament, the German “Tierschutzgesetz” (animal protection law) and approved by the local authorities (LANUV NRW: Landesamt für Umwelt, Natur und Verbraucherschutz Nordrhein Westfalen, Bezirksregierung Düsseldorf; permission number O58/91). Animal studies are reported in compliance with the ARRIVE guidelines. All efforts were made to minimize the number of animals and their suffering.

**Fig. 1:**
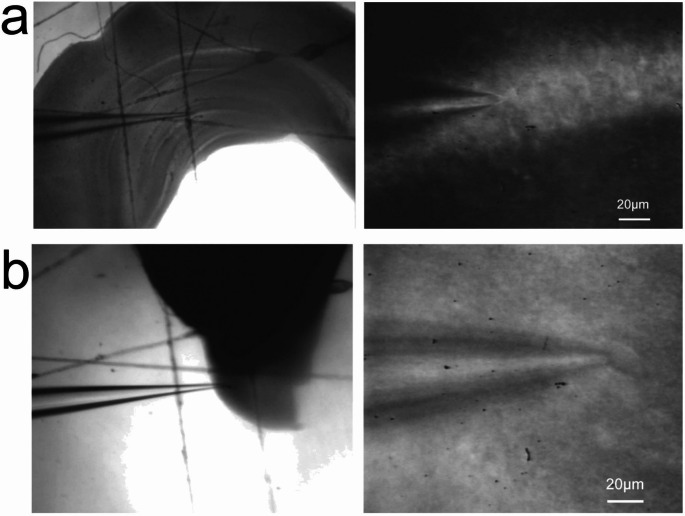
Representative photographs of the recorded cell and an overhead view of the slice containing this cell (fixed in the recording chamber and perfused with CF-ACSF at a speed of 2 ml/min) for** a** hippocampus and** b** TMNv. Images were obtained using either transmitted light (left) or an infrared light and differential interference contrast (IR-DIC) on the right

Coronal brain slices (250 μm thick) at the level of TMN, normally 2–3 per mouse, were prepared with a tissue slicer (HR2, Sigmann Elektronik, Germany) as previously described [19]. Tissue cutting was done in ice-cold artificial cerebrospinal fluid (ACSF) saturated with carbogen (5% CO_2_/95% O_2_) to maintain the pH at 7.4. Composition of cutting solution was (in mM): Sucrose (202), D-Glucose (12), NaHCO_3_ (26), KCl (3.75), NaH_2_PO_4_ (1.25), MgSO_4_ (1.3), CaCl_2_ (2). Further incubation steps were done in ACSF with 125 mM NaCl instead of 202 mM sucrose. Immediately after cutting and dissection of HPC and TMN-containing slices we transferred them to the incubation glass and ramped the temperature of ACSF from 14 to 22 C° to 32–34 °C during 30 min; after that slices were incubated at room temperature (~ 22 °C) all the time (1 to 7 h) before recording. A slice was transferred to the heated chamber (27–30 °C), where recording was started after an accommodation phase of about 30 min. Cells were visualized and approached under infrared light and differential interference (IR-DIC) optics (Axioscope 2 FS, Zeiss, Oberkochem, Germany). The image was detected with an infrared-sensitive video camera (Newvicon C2400; Hamamatsu, Hamamatsu City, Japan). The patch pipettes were made from 1.5 mm (OD) borosilicate glass (Science Products GmbH, Hofheim, Germany) using a horizontal microelectrode puller (P-87, Sutter Instruments) and filled with ACSF (resistance 4.5–6 MΩ). See examples of recorded cells in Fig. 1. To avoid polysynaptic effects of adenosine, recordings were done in calcium-free ACSF (CF-ACSF) perfused at 2 ml/min. In CF-ACSF Ca^2+^ was omitted and EGTA (0.5mM) was included. In addition, the MgSO_4_ concentration was increased to 2.6mM in order to allow stable long-term recordings.

We have shown previously that the switch from normal ACSF to CF-ACSF depolarizes HN and increases transiently their firing frequency [20], whereas upon long-term incubation in CF-ACSF, firing frequency of HN does not differ from control condition. In agreement with our previous study [20], in the present study basal firing frequency of HN in normal ACSF (0.9 ± 0.3 Hz) did not differ from the frequency in CF-ACSF (2.4 ± 0.5 Hz ) (MWT, *p* = 0.11, U(N1 = 25, N2 = 7) = 52). In cholinergic neurons of the neostriatum, calcium-depleted ACSF causes a reduction of the slow afterhyperpolarization (calcium-dependent potassium conductance) which may influence the firing pattern [21]. To exclude the possibility that the aforementioned conditions impaired adenosine-induced responses in HN, we performed some experiments in normal ACSF containing antagonists of GABA_A_- and glutamate- receptors (gabazine 10 µM, D-AP5 50 µM and CNQX 20 µM). Histaminergic neurons were recorded in the ventrolateral part of TMN (TMNv, Fig. 1b). To record DGgc, the tip of the electrode was positioned over the granule cell body layer in the Dentate Gyrus (DG) (Fig. 1a) and cells were approached one after the other with a waiting time for the spikes of 10–15 min after a good seal was established. If none of 10 to 15 patched cells showed spontaneous activity, the next slice was taken for recording. In agreement with previous studies we found that the vast majority of DGgc is silent [22], only few cells are spontaneously active in mouse brain slices [23]. Incubation of guinea pig hippocampal brain slices in ACSF with Ba^2+^ 1 mM / Ca^2+^ 0 mM resulted, in a study by Fricke and Prince [24], in the appearance of membrane depolarization waves with burst discharge in DGgc instead of silence under normal conditions. In our experiments the probability to detect a firing granular layer cell was somewhat higher in CF-ACSF. In experiments where DGgc were sequentially perfused with two different solutions, firing frequencies represented on average 3.5 ± 0.9 Hz in CF-ACSF and 0.47 ± 0.18 Hz in normal ACSF (*n* = 12, *p* = 0.0015, Wilcoxon matched pairs signed rank test), among those 12 cells, five became silent after switching from CF-ACSF to normal ACSF. Therefore, all experiments with adenosine application to DGgc were performed in CF-ACSF.

Histaminergic Neurons (HN) from wild type mice were considered for the analysis if they were inhibited by the H3-receptor agonist (R)(−)-α-Methylhistamine (RAMH, 2 µM) or excited by the H3-receptor antagonist clobenpropit (10 µM), which were usually applied at the end of experiment. Signals were recorded in cell-attached voltage clamp mode with the Multiclamp 700B amplifier (Molecular Devices, USA), they were filtered between 0.5 and 10 kHz, sampled at 20 kHz and analysed online using pClamp10 software (Axon Instruments, USA) in bins of 60 s duration. If during the pre-testing period (10 min) the frequency of firing was unstable or changed more than 2-fold, the cell was not further recorded. For the construction of time-course diagrams of each individual recording, extracellular action potential currents were detected and analysed with MiniAnalysis 6.0.1. (Synaptosoft inc, Fort Lee, NJ, USA). Interevent Intervals were collected for the whole recording period (in 60 s bins) and used for the statistical analysis. The experimental protocol included 7 min control, 7 min application of receptor agonist and 15 min washout periods. In case of co-application of agonist and antagonist our protocol was: 7 min control ACSF perfusion, 7 min antagonist application, 7 min common application of agonist and antagonist and 15 min washout in antagonist.

### Single-cell RT-PCR

DGgc and HN were harvested from slices either after recording or without recording and processed for single-cell RT (reverse transcription)-PCR (scRT-PCR) as previously described [25]. Briefly, electrode content (8 µl) was added to the 7 µl of the first-strand cDNA Synthesis kit mixture (GE Healthcare, GB), mixed and incubated overnight at 37 °C. Reverse transcription was terminated by freezing at -80 °C. The resulting cDNA samples were then used as templates for the two-round amplification strategy for histidine decarboxylase (HDC), PDZd2 (marker of dorsal DGgc) and A1R (for primers and reactions see Table 1). HDC- or PDZd2-negative samples were assigned as “PCR negative” and excluded from further amplifications.

**Fig. 2:**
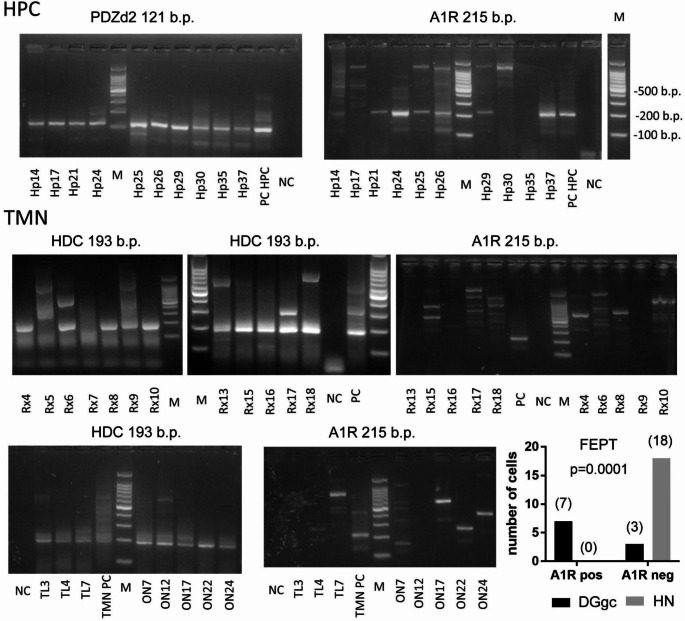
Gel electrophoresis photographs illustrate the results of scRT-PCR experiments. Only gels with PDZd2-positive cells shown. The numbers of A1R-positive (pos) and negative (neg) cells given in brackets in the plot at the bottom line. The probability of A1R detection in HN differs significantly from DGgc (FEPT: Fisher´s exact probability test)

All PCR reactions were performed in a volume of 10 µl, with cDNA template not exceeding 15% of the reaction. Results of PCR amplification were analysed with the help of 2.5% agarose gel electrophoresis (See Fig. 2) stained with “GelRed Nucleic Acid Gel Stain“ (Biotium, Hayward, CA, USA). Selected PCR products of expected size were purified in water and subjected to Sanger sequencing to verify specificity of mRNA amplification. All obtained sequences corresponded to the known mouse transcripts (Gene Bank NCBI ID): HDC (NM_008230), A1R (NM_001282945), PDZd2 (NM_001081064). Sequencing chromatograms assured mRNA amplification as exon/exon junctions were detected.

**Table 1:** Primers used for quantitative (Q)- or/and for single-cell RT- PCR amplifications (sequences are written in sense (forward) and antisense (reverse) direction) in the first (1) amplification round or in the second (2) amplification round

	forward	Reverse	size, b.*p*.
A1R (Q,2)	ACCTGCCTCATGGTGGCCTG	ACTCAGGTTGTTCCAGCCAAAC	215
A1R (1)	ACCTGCCTCATGGTGGCCTG	GTAGTACTTCTGGGGGTCACC	443
PDZd2 (2)	TGGTCATCAGCCAGGTGGAACT	GAGTCCAGTGTTTGGGTGGCAT	121
PDZd2 (1)	TGGTCATCAGCCAGGTGGAACT	GTCAGGACGAACTTCCCTGC	238
HDC (2)	GATGATGGAGCCCTGTGAATA	GATGCTGTCCCAGCTGTCG	193
HDC (1)	GATGATGGAGCCCTGTGAATA	TCAGAGGTGTAGGCAACGA	594
RpL13a(Q)	ATGACAAGAAAAAGCGGATG	CTTTTCTGCCTGTTTCCGTA	214

For each amplification, the expected PCR product size is given at the right side (in base pairs, b.p.)

### Semiquantitative PCR (qPCR)

The comparative ΔΔCt method, developed by Schmittgen and Livak [26], was used in the present study to analyze qPCR data. We amplified cDNAs derived from the whole caudal hypothalamus (cHPT) or hippocampus, both dissected from the coronal slice (0.5–1 mm thick) cut at the level of the tuberomamillary nucleus (TMN), in the Applied Biosystems StepOne real time PCR machine using the SYBR Green master mix kit (Applied Biosystems). Reactions were performed in MicroAmp optical 96-well plates in a total volume of 10 µl containing the final concentration of SYBR Green PCR Master mix, cDNA (100–150 ng) and primers. The following thermal protocol was used: initial incubation at 50 °C for 2 min to activate uracil N-glycosylase, 10 min at 95 °C to inactivate the uracil N-glycosylase and activate the AmpliTaq Gold Polymerase, and finally 40 cycles of 15s at 95 °C and 1 min at 60 °C. At the end of PCR cycling reactions were subjected to the heat denaturation protocol, all products showed a single peak in DNA melting curves. All reactions were carried out in duplicates. The relative mRNA level of A1R (in % of Mouse #2 HPC) was estimated by the “2^−ΔΔCt^“ method using normalization on the expression of the ribosomal protein L13A (RpL13a) [27, 28], where ΔCt = Ct target gene- Ct RpL13a. Standard curves were obtained with sequential dilutions of one cDNA sample (till 1:128) and were found optimal for both: A1R and RpL13a protocols (linear regression coefficients were > 0.95).

### Cultures and microelectrode array (MEA) recordings

Primary dissociated cultures from the whole hippocampus and posterior hypothalamus of mice aged 0 to 4 days (*n* = 2–6 per plating) were prepared in parallel fashion as previously described in Sergeeva et al. [15]; cells were dissociated and plated in a volume of 60 µl onto polyethylenimine (Sigma/Aldrich cat.(catalogue) No. P3143)-coated MEAs (cat. No. 890953, Multi Channel Systems, Reutlingen, Germany) in a minimal essential medium (MEM, Gibco cat. No. 11090-081)–based growth medium as in Sergeeva et al. [15], cells were cultured in an incubator with 5% CO2, 95% air and 98% relative humidity, at 37 °C. Four hours after plating the final volume of neurobasal medium (NBM plus)-based or MEM-based growth medium (1.6 ml per MEA), both supplemented with 2% B27 plus (Invitrogen cat. No. A35828) and 1% Penicillin-Streptomycin (Sigma/Aldrich, cat. No. P4458), was added to hippocampal or hypothalamic cultures, respectively. Medium was exchanged once a week. Extracellular potentials were recorded on MEAs with a square grid of 60 planar Ti ⁄ TiN-microelectrodes (30 μm diameter, 200 μm spacing). Signals from all 60 electrodes were simultaneously sampled at 25 kHz, visualized and stored using the standard software MCRack provided by Multi Channel Systems. Spike detection was performed offline using the software SpAnNer (RESULT Medizinische Analyseverfahren, Toenisvorst, Germany). Individually for each channel, the threshold for spike detection was set to eight standard deviations of the average noise amplitude during a 10% “learning phase” at the beginning of each measurement. An absolute refractory period of 4 ms and a maximum spike width of 2 ms were imposed on the spike detection algorithm. Spontaneous spike rate (Number of Spikes per minute, further referred to as Firing Frequency: FFR) was averaged over all electrodes. In all recorded MEAs SpAnNer (Analysis software) detected 10 or more active channels. Recordings were conducted from 11 independent platings after 6 to 24 days in vitro at temperature 33–37 °C in a HEPES-buffered solution (HBS) containing (in mM): NaCl, 150; KCl, 3.7; CaCl2, 2.0; MgCl2, 0.5; HEPES, 10, glucose 10, pH adjusted to 7.4. Before recording, the growth medium was replaced by HBS and measurements were started after a 10 to 20 min adaptation phase. Every measurement comprised 7 min baseline, 5 min application period and 10–15 min washout (second control) with non-interrupted recording in 60 s-long files (bins). FFR is presented as mean ± SEM, n refers to the number of recordings, N to the number of independent experiments (platings).

### Drugs

Drugs used for the electrophysiological experiments were obtained from Sigma-Aldrich Chemie GmbH (Taufkirchen, Germany): Adenosine (cat. No. A9251), 8-Cyclopentyl-1,3-dipropylxanthine (DPCPX, cat. No. C101), (R)(−)-α-Methylhistamine dihydrochloride (RAMH, cat. No. H128); from Tocris (Bio-Techne, Wiesbaden-Nordenstadt, Germany): clobenpropit (cat. No. 11213569); and from Abcam (Cambridge, UK): SR 95531 (gabazine, cat. No. ab120042), CNQX disodium salt (cat. No. ab144488), (2R)-2-amino-5-phosphonopentanoic acid (D-AP5, cat. No. ab120003).

### Statistical analysis

Data are presented as the mean ± SEM. Statistical analysis was performed using Excel and GraphPad Prism versions 5 or 7.08 (La Jolla, CA, USA). Analysis of variance method combined with appropriate posthoc tests was used for the comparison of more than 2 groups. If the Shapiro-Wilk normality test detected no normal distribution at least in one group, the Kruskal-Wallis test, followed by Dunn´s multiple comparisons test, was used (reported as H(df, N) = statistical value, where df: degree of freedom (number of groups-1), N: total number of values). We used the non-parametrical Mann Whitney U-test (MWT) for comparison between two groups, if no normal distribution of data was observed (statistical value reported as U(N1,N2), where N1: number of cells in group 1 and N2: number of cells in group 2), the unpaired t-test if data were normally distributed and had equal variances and the unpaired t-test with Welch´s correction for the comparison between two groups with normal distribution and unequal variances (results reported as t(df) and p values). For the comparison of response time-dependence between two brain regions, we used Two-way ANOVA with repeated measures (RM). The difference in effect probability was estimated with Fisher`s exact probability test (FEPT). A value of *p* < 0.05 was considered statistically significant.

## Results

### Probability of A1R transcript detection differs between DGgc and HN

In total, we harvested 26 DGgc and 24 HN for scRT-PCR (identified either pharmacologically by the response to (R)(−)-α-Methylhistamine (RAMH) or by red fluorescence in Tmt-HDC mice). A1R expression was examined in 18 HDC-positive cells (obtained from 9 mice) and in 10 PDZd2-positive DGgc (6 mice). In the remaining 16 DGgc, no PDZd2 transcripts were detected. A1R was detected in 70% of DGgc cells, whereas none of the HN were A1R positive (Fig. 2). Fisher ´s Exact Probability Test (FEPT) demonstrated a significant difference in the detection rate of A1R between HN and DGgc (Fig. 2, bottom).

### Hippocampal level of A1R mRNA is higher than hypothalamic level (qPCR)

Hippocampal and posterior hypothalamic tissues were dissected and analysed in parallel fashion in six male mice aged 230 ± 31 days. The qPCR showed that the hippocampus of adult mice expresses approximately 6 times more A1R compared to the posterior hypothalamus. Specifically, the mRNA levels comprised 105 ± 7% and 18 ± 2% in the hippocampus and posterior hypothalamus, respectively (t-test with Welch´s correction:

t (5.7) = 12.6, *p* < 0.0001).

### Adenosine inhibits DG granular layer cells

**Fig. 3:**
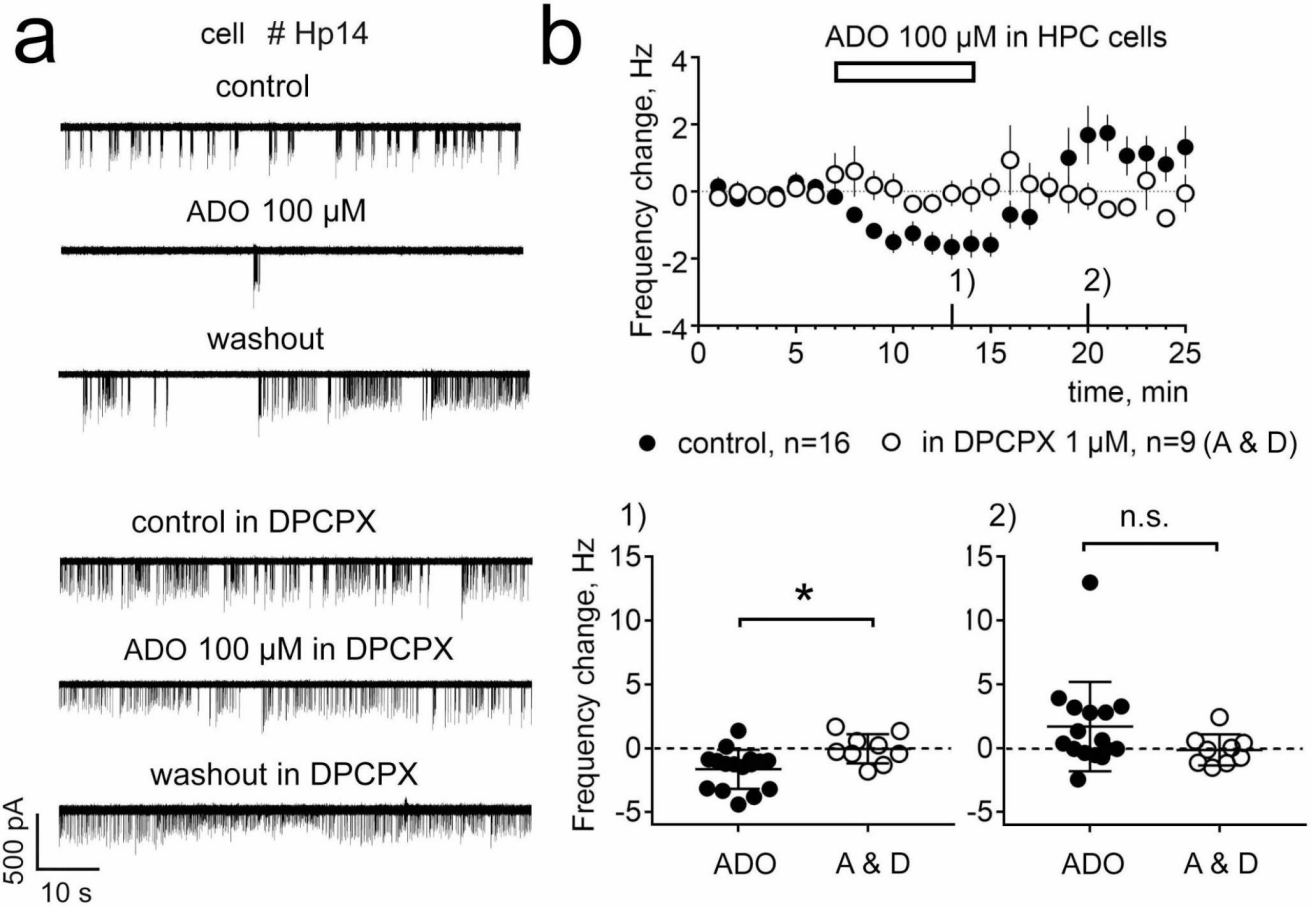
**a** An example of patch-clamp recording derived from PCR-positive DG granular layer cell # Hp14 (“good responder” to adenosine (ADO));** b** The average ± SEM of firing frequency change from 7 min average of baseline versus time (data from all recorded DGgc). Individual data points below for the 13th min of experiment (1) and 20th min of experiment (2). Results of unpaired t-test (t (23) = 2.7, *p* = 0.012) for (1) (both groups are characterized by normal distribution according to Shapiro-Wilk test) and of Mann-Whitney U-test (U(N1 = 16,N2 = 9) = 41, *p* = 0.08) for (2) (no normal distribution in “ADO” group) are indicated above the plots

The majority of recorded DGgc (Fig. 3) showed a reduction in their firing frequency (FFR) by 1.6 ± 0.36 Hz (*n* = 16) in response to adenosine at 100 µM. Repeated measures one way ANOVA followed by the Dunnett´s multiple comparisons test showed a significant difference (F(2,30) = 8.5, *p* = 0.0012) between baseline and application period (*p* < 0.01) and no difference between baseline and washout period (0.27 ± 0.48 Hz (*n* = 16)). In 6 cells where the response to 100 µM adenosine was clear and FFR recovered to the baseline level within 10 min during the washout period, the second application of 100 µM adenosine yielded a response (FFR reduction by 1.3 ± 0.35 Hz) not significantly different from the first one (FFR reduced by 0.7 ± 0.25 Hz; paired t-test, *p* = 0.3; t(5) = 1.149). Next, we performed a second adenosine application in such “good responders” in the presence of the A1R antagonist DPCPX. Comparison with control experiments showed a significant impairment of the response to 100 µM adenosine (FFR change: 0.14 ± 0.4 Hz, *n* = 9) in the presence of the antagonist (t(23) = 2.99, *p* = 0.0064). Application of 10 µM adenosine in “good responders” always resulted in a smaller response (50.1 ± 11% of the response to 100 µM adenosine, *n* = 5). Therefore, in our experiments with HN, we used an adenosine concentration of 100 µM.

### The majority of HN is not inhibited by adenosine

**Fig. 4:**
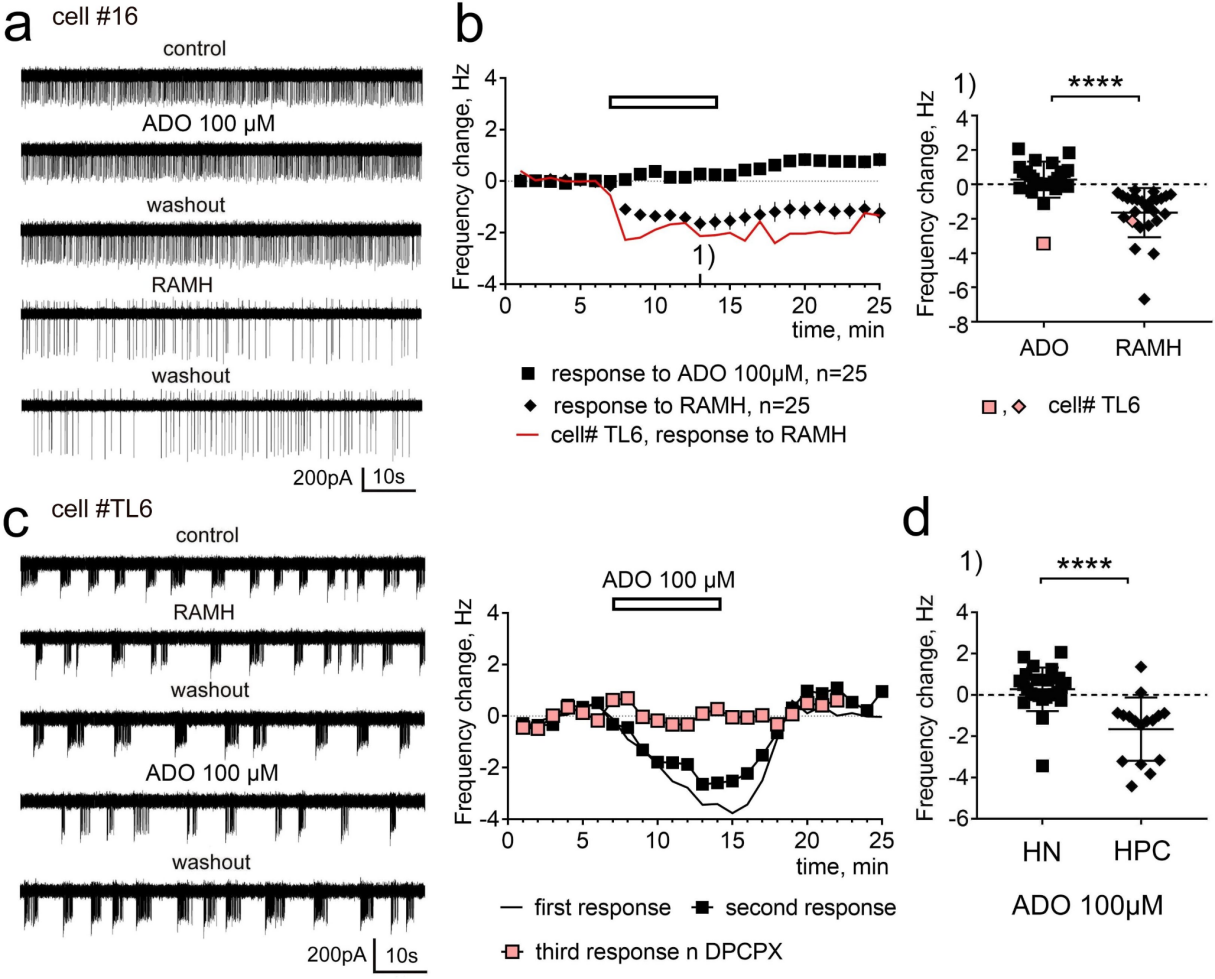
Examples of patch-clamp recordings from HN and averaged time course diagrams of responses to (R)(−)-α-Methylhistamine (RAMH) and adenosine (ADO). **a** Recordings derived from HN #16. **b** Left: time course diagrams (mean + SEM) of firing frequency change by RAMH and adenosine in 25 recorded HNs. Response to RAMH in cell # TL6 is shown by line without symbols. At the right side individual data points for the 13th minute of recordings (1) as indicated in left plot are shown. MWT U(N1 = 25, N2 = 25) = 36, *p* < 0.0001 (****). **c** Examples of recorded current traces from cell # TL6. Note “burst-like” firing patterns atypical for HNs. Time course diagrams of frequency change show responses to repetitive applications of adenosine in this cell. **d** Comparison of individual data points for the 13th minute of recording (as indicated by (1) in Fig. 3 and in panel **b**) between HPC and TMNv HN reveals significant difference (MWT U(N1 = 25, N2 = 16) = 50, *p* < 0.0001)

In patch-clamp experiments, one HN out of 25 (20 mice) was inhibited by adenosine reliably several times, but not in the presence of DPCPX (A1R antagonist) (Fig. 4, cell# TL6). Repeated measures (RM) one way ANOVA followed by the Dunnett´s multiple comparisons test showed no significant difference between baseline and application period (0.22 ± 0.15 Hz (*n* = 25)), but a significant difference between baseline and washout (average of 16th to 20th minute of experiment) period (0.62 ± 0.18 Hz (*n* = 25), *p* < 0.001). RM one way ANOVA revealed significant individual (cell to cell) differences (F(24,48) = 3.231, *p* = 0.0003) and effect of the treatment (F(2,48) = 9.04, *p* = 0.0005). Thus, adenosine increased firing of HN during post-application period, but did not change it during application. Two-way RM ANOVA revealed significant difference between responses to adenosine in DG granular layer cells and HN (F(1,39) = 10.04, *p* = 0.003).

In order to exclude possible influence of calcium-free condition on responsiveness of HN to adenosine, we performed additional experiments in normal ACSF in the presence of gabazine (10µM), CNQX (20µM) and D-AP5 (50µM), further called “ACSF + GCA” (7 neurons, 5 mice). Experimental results obtained in two different extracellular solutions were similar. Frequency change during 7 min of adenosine application amounted to 0.17 ± 0.18 Hz and during washout period (16th to 20th min of experiment) to 0.37 ± 0.2 Hz in ACSF + GCA.

Two-way RM ANOVA revealed no difference between responses to adenosine in HN recorded in CF-ACSF vs. ACSF + GCA (F(1,30) = 0.24, *p* = 0.6).

### Cultured hypothalamic neurons are less sensitive to adenosine than hippocampal neurons

**Fig. 5:**
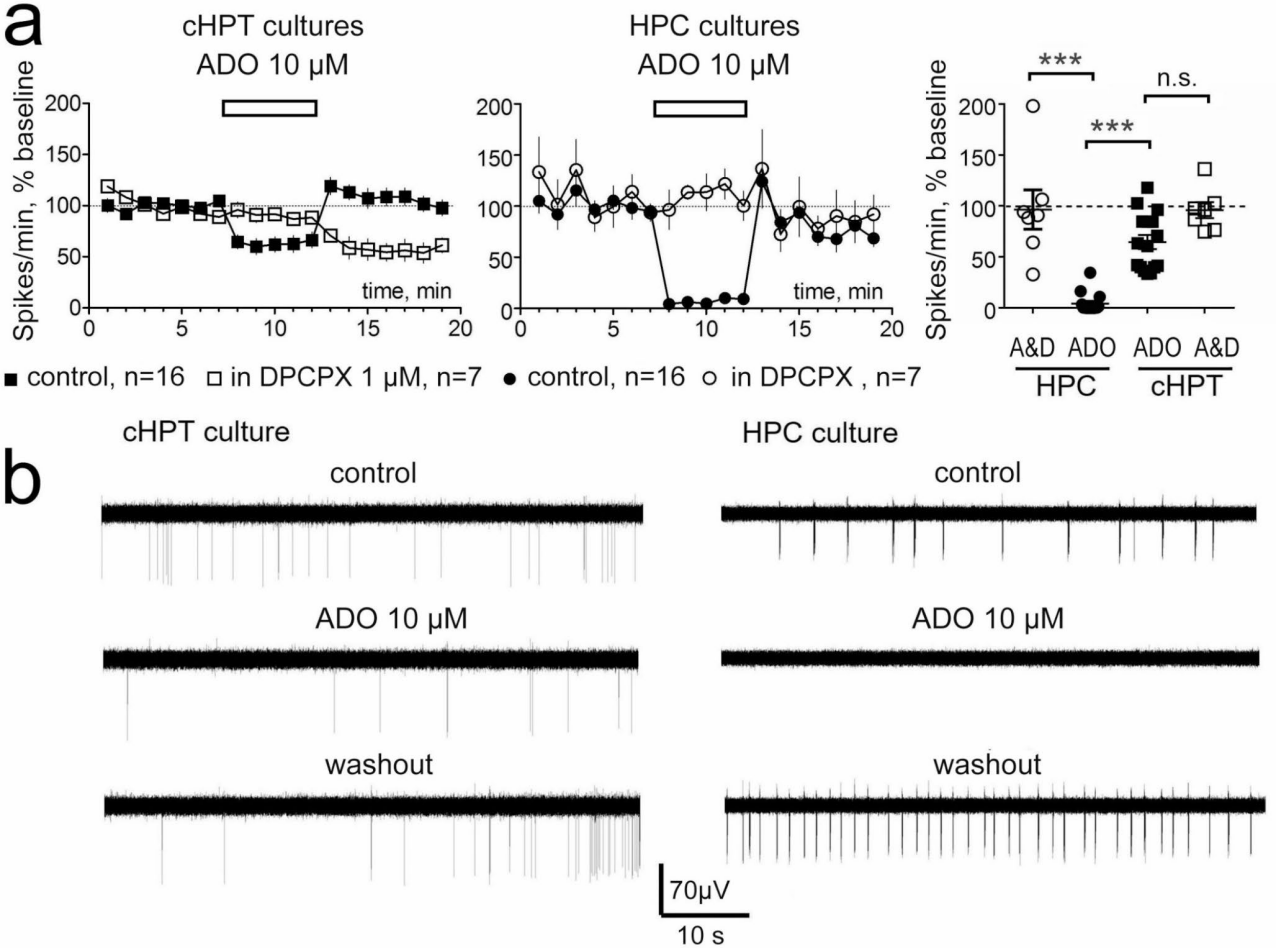
MEA recordings from cultured neurons. **a** Averaged time-course diagrams show total number of spikes per minute (Spike/min) as percent of 7 min baseline (Mean ± SEM) with adenosine (ADO) application for 5 min followed by the washout period (control recordings, black symbols) or recording in the presence of DPCPX (open symbols) in cHPT and HPC cultures left and middle plots, respectively. Right plot shows results of Kruskal-Wallis test followed by Dunn´s multiple comparisons test performed for the individual data points of the first minute of adenosine application period in 4 experimental sessions.***: *p* < 0.0005. **b** examples of MEA recordings from cHPT and HPC cultured neurons

In primary dissociated cell cultures of the hippocampus (HPC), the inhibition of the firing rate in the first minute after adenosine application was significantly stronger than in cultures derived from the caudal (posterior) hypothalamus (cHPT) (Kruskal Wallis Test (H(3,46) = 32.68, *p* < 0.0001), Dunn’s multiple comparisons test, *p* < 0.0005). Relative to baseline FFR was reduced to 4.3 ± 2.4% in the HPC (*n* = 16, *N* = 9) and 64.6 ± 7% in the cHPT (*n* = 16, *N* = 7) (Fig. 5).

Because A1R desensitization and internalization are well studied in neuronal cultures [29], we tested the effects of repeatedly applied adenosine in the same recordings. A second adenosine application did not induce a significantly different decrease in FFR (0.97 ± 0.47% of baseline) compared to the first application in HPC (3.15 ± 2.2% of baseline, *n* = 7, *N* = 5), paired t-test (t(6) = 1.2, *p* = 0.27). This allowed us to apply adenosine a second time in the presence of DPCPX. The response to 10 µM adenosine was significantly attenuated by DPCPX 1 µM in the HPC (*n* = 7, Kruskal Wallis Test, Dunn’s multiple comparisons test *p* < 0.0005) but not the cHPT (*n* = 7, Kruskal Wallis Test, Dunn’s multiple comparisons test *p* > 0.05). In the presence of DPCPX, FFR relative to baseline comprised 96.5 ± 19.3% in the HPC (*n* = 7, *N* = 5) and 95.9 ± 7.8% in the cHPT (*n* = 7, *N* = 5) during the first minute of adenosine application.

## Discussion

The main finding of the present study is the significant difference in expression of A1R between HPC and cHPT and between DGgc and HN in responsiveness to adenosine and A1R expression. Both cellular types were recorded under the same condition in CF-ACSF, which increases cellular excitability and the probability to detect firing cells. This is the first study to our knowledge where positive control (DGgc) was studied in parallel to HN of TMNv. Our experimental design allowed us to select an application/washout protocol to obtain high reproducibility of response within one experiment on DG granular layer cell, where responses of the same amplitude were seen upon repeated applications. When this protocol was applied to HN, only one cell out of 25 was significantly and repeatedly inhibited by adenosine. Further 7 HN (5 mice) were recorded in normal ACSF supplied with gabazine, CNQX and D-AP5 to block synaptic inputs and to exclude the possibility of impaired responsiveness to adenosine without extracellular calcium. No difference in HN responsiveness to adenosine was detected between two recording solutions. Responding to adenosine in CF-ACSF cell (# TL6) turned out to be PCR-negative and was only identified by the response to RAMH (H3R agonist). A recent study published single-cell whole transcriptome analysis data [9] from 22 populations of caudal hypothalamic neurons and found, that in addition to HN (cluster #17), two other (GABAergic) populations of neurons express H3R: namely Nucleus Arcuatus NPY/AGRP neurons, which are located around the third brain ventricle and lateral hypothalamic somatostatin-positive GABAergic cells (cluster #19) with diffuse distribution in the hypothalamus. All three clusters (#17, #19, #20) are expressing some overlapping markers, such as H3R, MaoA and Bcl2. We can exclude the possibility that we recorded a NPY/AGRP neuron (cell#TL6) as our recordings were performed from ventrolateral TMN (TMNv) (see Fig. 1), located near the lateral surface of the brain. We cannot exclude, however, that the cell #TL6 belongs to cluster #19. In none of the neuronal clusters described in the study by Mickelsen et al. [9] A1R was detected. It is a common limitation of single-cell RNAseq data that mRNAs encoding for receptors and ion channels often remain below detection level. In-situ hybridization databases (Allen-Mouse-Brain-Atlas, Lein et al. [30]) also do not reveal a notable A1R-expression in the hypothalamus but do so in the hippocampal formation. A study from 1992 employing autoradiography for DPCPX-H3-binding demonstrated that A1R density in the hypothalamus of the rat is 8 times lower compared to the hippocampus [31]. This is in line with our publication in 2006, where none of rat HN were responding to adenosine and no response to adenosine was recorded in primary dissociated hypothalamic cultures. Unfortunately, “no response” may mean pharmacological artefact, if no positive control is given. The study by Oishi et al. [3] showed immunohistochemistry for A1R colocalized in mouse TMN with HDC: this raised doubts whether our study in 2006 was done correctly. Our present study was designed based on recently published whole transcriptome data of hippocampal and hypothalamic neurons and allowed us to identify the positive control, which helped to develop time and scRT-PCR protocols where HN cells were processed in parallel with positive control: DGgc. This demonstrated that, in contrast to DGgc, HN do not express a detectable level of A1R. Did we harvest cells at the peak of circadian A1R expression? “CircaDB” database [32] shows that the peak of A1R expression corresponds to the sleep period in rodents (light-on phase). During this period, we harvested cellular cytoplasm from the brain slices. This expressional peak puts doubts on the role of A1R for sleep onset under normal conditions, but it is in accordance with the proposed function: to report about sleep need during sleep deprivation [7]. In humans A1R density increases after sleep deprivation (SD) and returns to baseline after recovery from SD [33, 34]. Interestingly, A1R knockout mice do not show impairments in the sleep-wake cycle or in EEG power [35], whereas Adenosine A2A receptor (A2AR) knockout mice show reduced slow wave sleep [36]. Accordingly, the peak in the circadian rhythm of this gene expression (A2AR) [32] corresponds to sleep onset - end of the night activity period - in nocturnal animals, which allows to assign to this receptor a role in sleep-wake regulation by adenosine [7].

How can controversial findings from previous studies [2–5] on TMN HN regarding expression and function of adenosine receptors be interpreted? First, evidences were mainly obtained in these studies by different methods without clear positive and negative controls. Second, data on diversity within the HN population and among hypothalamic neurons 15 years ago were not yet available. In addition, taking into consideration that electrophysiology detects receptors with higher sensitivity compared to the transcriptome analysis, combination of all possible approaches is nowadays needed. In addition to HN, a role of astrocytes [37–39] and GABAergic afferents of HN [4, 5] need to be considered. GABAergic inputs of HN were found to be modulated by adenosine either via A2AR [4] or via A1R [5]. We used in our study CF-ACSF to avoid polysynaptic and presynaptic effects and to isolate postsynaptic responses. Diversity of GABAergic inputs to HNs awaits further analysis in the future. Based on discovery of cell-type specific markers (published in recent whole-transcriptome studies) it will be possible with the help of reporter mouse lines to study separately different populations of GABAergic neurons of lateral hypothalamus and lateral preoptic area.

Adenosine-Deaminase (ADA), an adenosine-degrading enzyme, is highly expressed in the TMN with the enzyme activity around 12 times higher in the posterior hypothalamus compared to the Dentate Gyrus [40]. In the rat, an antibody against ADA labels the same population of neurons in TMN as an antibody against HDC [41]. In our previous study in the rat brain slice preparation of TMN [2], we did not see effects of the ADA inhibitor EHNA (erythro-9-(2-hydroxy-3-nonyl) adenine hydrochloride) 20µM, applied either alone or in combination with adenosine 200µM. We used low recording temperature in our slice experiments (27–30 °C), which, in studies by Masino et al. [42] and Takahashi et al. [43] did not allow endogenous release of adenosine from hippocampal slices and spinal cord preparations in contrast to 37 °C or higher temperature. Different degradation and release pathways of adenosine and their difference between DG and TMN are beyond the scope of the present study.

Electrophysiology of brain neurons recorded in slices is not identical to the cellular properties recorded in vivo. Previous studies have reported that DGgc recorded in brain slices have less inhibitory inputs compared to the situation in vivo, which can be explained by the damaged longitudinal ramifications of the interneurons [23]. Moreover, we used in our recordings CF-ACSF which depolarizes cells and enhances their excitability [20]. This should be considered regarding our data reporting firing patterns. We cannot exclude, without cellular labelling or successful scRT-PCR in every cell, the possibility of occasional recording from DG granular cell layer interneurons, which are actually rarely preserved in slices. As adenosine levels increase in response to hypoxia and cellular damage [43, 44]– conditions that are likely present during tissue slicing and maintenance - we performed recordings also from cultured neurons which remained intact before, after and during the experiment. In both preparations, we saw lower sensitivity of hypothalamic neurons to adenosine compared to hippocampal neurons. Sensitivity to adenosine of cultured HPC neurons was about 10 times higher compared to the neurons recorded in brain slices (maximal response achieved by 10 µM vs. 100 µM, respectively). The same concentrations of adenosine, which we found to be effective in slices, were used in previously published studies [45, 46].

In conclusion, our study sheds more light on the role of A1R for the physiology of HN and DGgc. The same recording conditions and the same protocols for scRT-PCR and qPCR for HN and DGgc allowed us to conclude that spontaneous activity of HN is not reduced by adenosine. This is in contrast to GABA-mediated inhibition and ubiquitous GABAA receptor expression by HN [15–17].

## Conclusions

We conclude that the vast majority of HN, recorded in slices, is not inhibited by adenosine. These data suggest that low (relative to HPC) expression of A1R in the hypothalamus and irresponsiveness of wake-promoting neurons to adenosine may permit fight or flight responses: to remain awake as long as necessary despite of adenosine accumulation.

## Data Availability

Data is provided within the manuscript.
